# Neuronal Dysfunction Associated with Cholesterol Deregulation

**DOI:** 10.3390/ijms19051523

**Published:** 2018-05-19

**Authors:** Annalisa Marcuzzi, Claudia Loganes, Erica Valencic, Elisa Piscianz, Lorenzo Monasta, Sabrine Bilel, Roberta Bortul, Claudio Celeghini, Marina Zweyer, Alberto Tommasini

**Affiliations:** 1 Department of Medicine, Surgery and Health Sciences, University of Trieste, 34149 Trieste, Italy; elisa.piscianz@burlo.trieste.it (E.P.); bortul@units.it (R.B.); zweyer@units.it (M.Z.); 2 Institute for Maternal and Child Health-IRCCS “Burlo Garofolo”, 34137 Trieste, Italy; claudia.loganes@burlo.trieste.it (C.L.); erica.valencic@burlo.trieste.it (E.V.); lorenzo.monasta@burlo.trieste.it (L.M.); alberto.tommasini@burlo.trieste.it (A.T.); 3 Cluster in Biomedicine (CBM scrl), 34149 Trieste, Italy; Sabrouna14@live.com; 4 Department of Life Sciences, University of Trieste, 34128 Trieste, Italy; cceleghini@units.it

**Keywords:** mitochondria, neurons, apoptosis, autophagy, cholesterol pathway

## Abstract

Cholesterol metabolism is crucial for cells and, in particular, its biosynthesis in the central nervous system occurs in situ, and its deregulation involves morphological changes that cause functional variations and trigger programmed cell death. The pathogenesis of rare diseases, such as Mevalonate Kinase Deficiency or Smith–Lemli–Opitz Syndrome, arises due to enzymatic defects in the cholesterol metabolic pathways, resulting in a shortage of downstream products. The most severe clinical manifestations of these diseases appear as neurological defects. Expanding the knowledge of this biological mechanism will be useful for identifying potential targets and preventing neuronal damage. Several studies have demonstrated that deregulation of the cholesterol pathway induces mitochondrial dysfunction as the result of respiratory chain damage. We set out to determine whether mitochondrial damage may be prevented by using protective mitochondria-targeted compounds, such as MitoQ, in a neuronal cell line treated with a statin to induce a biochemical block of the cholesterol pathway. Evidence from the literature suggests that mitochondria play a crucial role in the apoptotic mechanism secondary to blocking the cholesterol pathway. Our study shows that MitoQ, administered as a preventive agent, could counteract the cell damage induced by statins in the early stages, but its protective role fades over time.

## 1. Introduction

The biosynthesis of cholesterol represents a fundamental pathway for the homeostasis of cells, as witnessed by the heterogeneous spectrum of disorders arising from genetic deficiencies of enzymes involved in this pathway, ranging from inflammatory disorders to complex neurodevelopmental diseases [1,2,3].

In general, the pathogenic mechanism of these disorders involves the shortage of compounds downstream of the enzymatic defect, rather than an accumulation of toxic molecules [2,4].

Indeed, although cholesterol is the end stage product of this pathway, several intermediate compounds can also contribute to the regulation of crucial cellular functions [1]. Moreover, the synthesis of these molecules is finely regulated in each cell type, and it is critical in the central nervous system where almost all cholesterol supply depends on in situ biosynthesis [5].

In particular, defective synthesis of cholesterol can affect the development of neural cells, and this deficiency may help to explain the occurrence of neurological symptoms in disorders of the cholesterol pathway [2], as Mevalonic Aciduria (MIM#610377) [6] and Smith–Lemli–Opitz Syndrome (MIM#270400) [7].

To date, we know that a common consequence of distinct alterations of the cholesterol pathway is represented by the triggering of programmed cell death, but the exact mechanisms underlying this effect are unclear [8,9]. Extending our knowledge of this biological process will be useful to identify potential cellular targets for medications aimed at preventing neuronal damage [10].

In previous studies, we demonstrated that blocking the cholesterol pathway in neuronal cells induces dramatic morphological changes to mitochondria, and we hypothesised that mitochondria play a major role in the induction of programmed cell death [8,11]. In order to mimic the blockade of the mevalonate pathway to study its molecular mechanisms at the mitochondrial level, our group developed a biochemical model applicable to several cell lines, including the neuronal one. This method identifies statins (lovastatin or simvastatin) as blocking systems of the same metabolic pathway that cause an increase in inflammatory markers [8].

Other authors recently proposed that mitochondrial damage by distinct causes may be prevented by protective mitochondrial-targeted compounds, such as MitoQ, MitoTempo, and MitoVitE [12,13].

In particular, the MitoQ, a ubiquinone similar to Coenzyme Q10, intervenes at the mitochondrial level limiting the effects of oxidative damage produced by free radicals released in the cellular respiration process, reducing inflammation and increasing the neuronal energy of the affected cells.

Although its mechanism of action is not fully known, some studies have indicated that MitoQ accumulates selectively in the mitochondria and not in the cells in general and is more effective as an antioxidant.

In vivo studies have shown that MitoQ rapidly enters neuronal mitochondria, destroys free radicals, reduces oxidative insults produced by high inflammation, and maintains or even increases neuronal energy in the affected cells. In recent years, this product has been considered promising for the treatment of neurodegenerative diseases such as Alzheimer’s disease and Parkinson’s disease [13].

**Figure 1:**
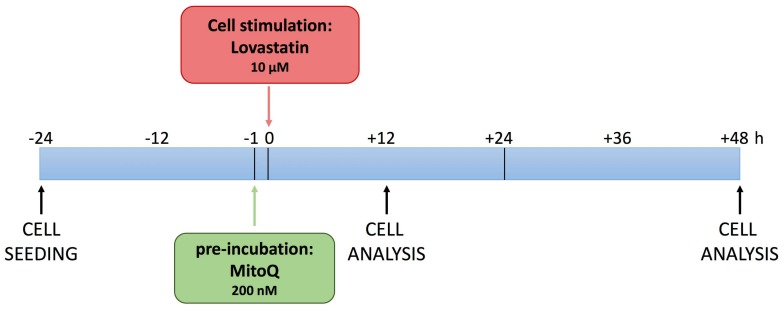
Schematic picture of the experimental design. DAOY cells were seeded (−24 h) and the pre-treatment of MitoQ was administered 1 h before lovastatin stimulation (0 h). Cell analyses were conducted after 12 and 48 h.

Based on these considerations, we investigated the potential of MitoQ in a neuronal cell line, DAOY, treated with lovastatin, as previously described [8], to induce a biochemical block of the cholesterol pathway. The experimental design allowed us to observe the functional and morphological changes induced by biochemical manipulation (Figure 1).

## 2. Results and Discussion

### 2.1. MitoQ Effect on the Impedance Profile

**Figure 2:**
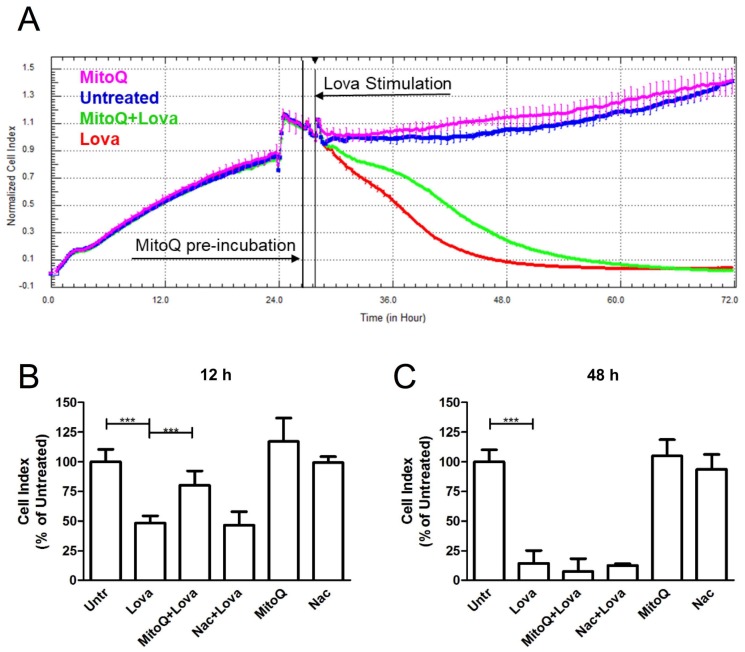
Real-time, label-free impedance analysis. (**A**) Representative impedance graph that shows the effect of lovastatin treatment and the potential protective actions of MitoQ added 1 h before the statins (pink: MitoQ; blu untreated; green Mitoq + Lova; red Lova). (**B**,**C**) DAOY cells were pre-incubated with MitoQ or *N*-acetylcysteine (Nac) and then stimulated with statins to evaluate the cell index at 12 (**B**) and 48 (**C**) h. Results are expressed as means and SDs in percentages (%), normalised to the control condition (Untr). (*n* = 3, *** *p* < 0.0001 based on a one-way ANOVA, Bonferroni post-test).

As shown by the representative impedance graph (Figure 2A), lovastatin (red line) induced a cytotoxic effect on cultured cells, proven by a decrease in the cell index (CI), a parameter derived by measuring cell status (Figure 2B) [14,15]. Pre-treatment with MitoQ, 1 h before lovastatin (green line), reduced the adverse effects linked to statin treatment—its protective actions occurred in the first 12 h after cell stimulation and tended to fade as time went by (Figure 2B). MitoQ alone (pink line) showed a line course that was comparable to the one of the untreated condition (blue line).

Lovastatin induced a statistically significant difference in normalized CI values when compared to untreated cells, both at 12 h and at 48 h (12 h: Untr: 100.1 ± 10.47 vs. Lova: 48.45 ± 5.92, *p* < 0.0001; Lova: 48.45 ± 5.92 vs. MitoQ + Lova: 80.17 ± 12.11, *p* < 0.0001) (48 h: Untr: 100 ± 10.01 vs. Lova: 14.56 ± 10.79, *p* < 0.0001).

Interestingly, the effect of MitoQ seems not to be limited to its global antioxidant action, since treatment with another potent and widely recognized ROS scavenger, *N*-acetylcysteine (Nac), failed to protect DAOY cells against cell death due to statins (Figure 2C) [16] (12 h: Nac + Lova: 46.67 ± 11.41 vs. Lova: 48.45 ± 5.92, ns; 48 h: Nac + Lova 12.83 ± 1.17 vs. Lova 14.56 ± 10.79, ns) (Figure 2B,C).

MitoQ and Nac, when administered alone, showed a trend similar to the untreated condition in both timings (12 h: MitoQ: 117 ± 19.81; Nac: 99.33 ± 5.16; Untr: 100.1 ± 10.47; ns) (48 h: MitoQ: 105.1 ± 13.48; Nac: 93.67 ± 12.53; Untr: 100 ± 10.01; ns) (Figure 2B,C).

The results obtained at 12 h show the protective and antioxidant effects of MitoQ very strongly, and this evidence includes a decrease at 48 h after stimulation. The results obtained with *N*-acetylcysteine highlight the specific effects of MitoQ that are able to prevent the modification induced by the block of the mevalonate pathway.

The observation through Xcelligence from MitoQ-preincubation to Lovastatin stimulation allowed us to choose the two time points to represent the extremes of the morphological and functional cellular changes of the effect of MitoQ for their optimal characterisation. The analyses carried out at intermediate times confirmed the change in cell morphology/function or adhesion strength and did not contribute to providing significant data.

### 2.2. Lovastatin Induced Lipid Accumulation

**Figure 3:**
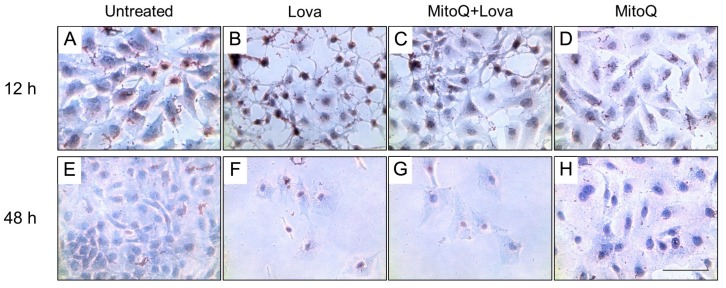
Representative light micrograph of Oil Red O staining in the DAOY cells. Neuronal cells were cultured for 12 h (upper panels) and 48 h (lower panels) with lovastatin, and with or without MitoQ. (**A**,**E**: Untreated condition; **B**,**F**: Lovastatin treatment alone; **C**,**G**: Lovastatin treatment associated with MitoQ; **D**,**H** MitoQ treatment alone). Scale bar 10 µm.

To establish the morphological modifications between 12 and 48 h after lovastatin treatment and the potential effect of MitoQ, we performed a first qualitative analysis using Oil Red O staining for lipids (Figure 3) [17,18].

The untreated condition, regardless of the time of analysis, showed optimum confluency. A compact cell monolayer, much more evident at the 48 h analysis, was observed—this condition did not appear to be altered by the presence of MitoQ in the culture media (Figure 3A,D,E,H) [19].

At 12 h after the lovastatin treatment, it was possible to observe disaggregation of the cellular layer and the formation of little clusters, signs of cellular distress, which was offset by the presence of MitoQ (Figure 3B,C). The dramatic effect of the blocking of the cholesterol pathway was highlighted 48 h after the statin treatment and did not recover with the addition of MitoQ (Figure 3F,G).

The Oil Red O staining allowed the lipid droplets that developed in DAOY cells treated with lovastatin to be highlighted (Figure 3) through an optical micrograph of neuronal culture stained with Oil Red O staining which selectively stained lipid droplets in the cells in red colour.

The block of the mevalonate pathway promoted the accumulation of Oil Red O stained lipid droplets in neuronal cells (Figure 3B,F). This effect was observed independently of the time of incubation but, in terms of cell density, the lipid droplet formation was more easily identifiable at 12 h compared to 48 h. (Figure 3B,F). At 12 h, MitoQ was able to counteract this lipid droplet formation (Figure 3C), and its effect suggests that the physiological function of the mitochondria is involved in the metabolism of cholesterol.

### 2.3. MitoQ Is Able to Reduce Programmed Cell Death Induced by Statin Treatment

**Figure 4:**
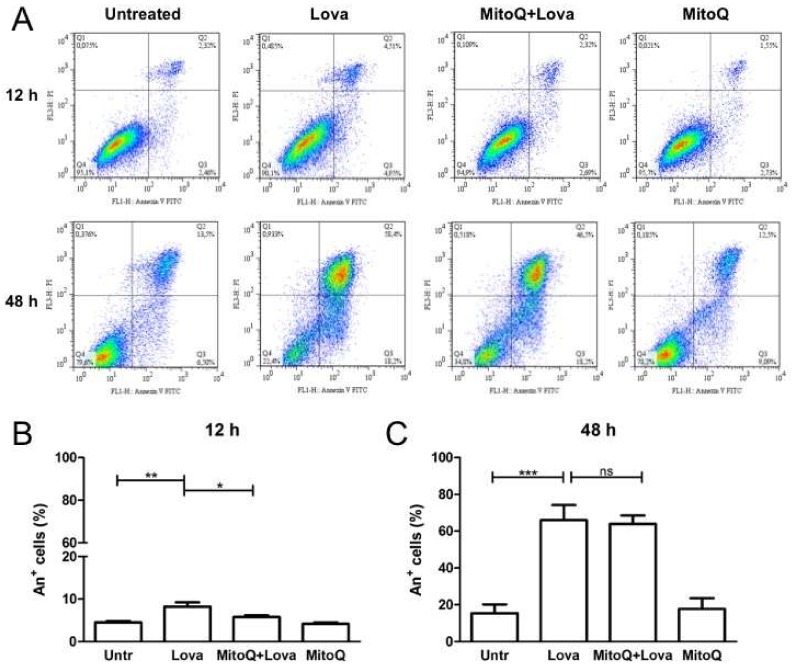
Programmed cell death of Lova-treated DAOY cells in the presence of MitoQ. (**A**) Representative cytofluorimetric dot-plots of Annexin propidium iodide staining in all experimental conditions at 12 h (**upper** panel) and 48 h (**lower** panel). (**B**,**C**) The results (A/P) obtained as means ± SDs of A+ cells of three independent tests at 12 h (**B**) and 48 h (**C**) (*n* = 3, ns: not significant; * *p* < 0.05; ** *p* < 0.001,*** *p* < 0.0001 based on a one-way ANOVA with Bonferroni post-test).

The harmful effect of lovastatin on DAOY cells was confirmed with the programmed cell death (PCD) analysis, assessed by the Annexin V-FITC (A+) Apoptosis Detection Kit. As expected, statin-treated cells were characterised by a major percentage of Annexin V positive cells, mostly at 48 h post-stimulation (Figure 4A: a vs. b; e vs. f) (12 h: Untr: 4.50 ± 0.68 vs. Lova: 8.207 ± 1.71, ** *p* < 0.001) (Figure 4B) (48 h: Untr: 15.39 ± 10.86 vs. Lova: 65.96 ± 18.47, *** *p* < 0.0001) (Figure 4C). The administration of MitoQ reduced the percentage of death cells at 12 h post-stimulation [20] (Figure 4A: c vs. b; g vs. f) (12 h: MitoQ + Lova: 5.78 ± 0.82 vs. Lova: 8.207 ± 1.71, * *p* < 0.05) (Figure 4B) (48 h: MitoQ + Lova: 63.92 ± 10.37 vs. Lova: 65.96 ± 18.47; ns) (Figure 4C). The MitoQ alone did not perturb cell viability, neither at 12 h, nor at 48 h.

The results highlight that 12 h of MitoQ pre-incubation led to a statistically significant decrease in PCD. This protective effect induced by MitoQ was gradually lost at 48 h after lovastatin stimulation. MitoQ, indeed, was not able to protect PCD in the long term.

Thus, the potential protective role of MitoQ in counteracting programmed cell death induced by statin was not so evident in later stages. The results of the analyses at intermediate times (24 and 36 h) are reported in the Supplementary Data (Figure S1).

### 2.4. MitoQ Reduces Morphological Changes Correlated with Deregulation of the Mevalonate Pathway

**Figure 5:**
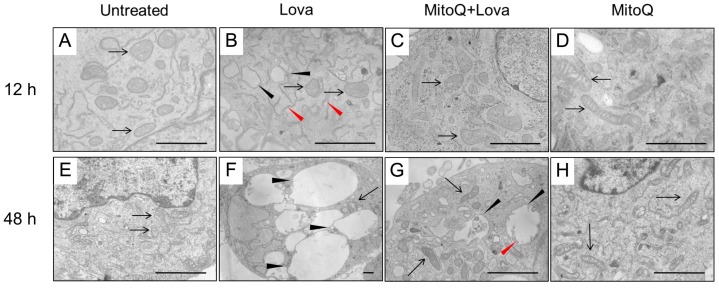
Morphological and functional changes associated with the cholesterol pathway block. Representative electron micrographs of DAOY cells incubated with lovastatin in the presence or absence of MitoQ, for 12 h (upper panel) and for 48 h (lower panel), showing the untreated condition (**A**,**E**), lovastatin treatment alone (**B**,**F**), and associated with MitoQ (**C**,**G**) and MitoQ alone (**D**,**H**). 12 h; (**A**) mitochondria are mainly oval in shape, exhibit regular membranes and cristae (arrows). (**B**) Mitochondrial cristae are not well defined (arrows). The cytoplasm shows vesicles that appear like empty mitochondria (black arrowheads). RER cisternae arranged themselves in the three space directions among mitochondria (red arrowheads). (**C**) Cell conditions improved much more compared to the lovastatin treatment alone. Most mitochondria were normal in shape (arrows). (**D**) Ultrastructure features are quite similar to untreated cells. Mitochondria are well-shaped, and the elongated shape is the most represented in these cell cultures (arrows). 48 h: (**E**) Mitochondria exhibit regular shapes, membranes and cristae (arrow). (**F**) Several very large vesicles are present in the cytoplasm (arrows). Some mitochondria show damaged cristae and clear areas in the matrix (arrowheads). (**G**) Large autophagic vesicles are evident (arrowheads). A mitochondrion is detectable inside a vesicle (arrow). Cytoplasmic mitochondria are normally shaped (red arrow). (**H**) Ultrastructure features are quite similar to untreated cells. Mitochondria are well-shaped. They appear a little more elongated compared to untreated cells (arrows). Bars = 3 micron.

To understand the role of mitochondria and other cellular districts involved in the block of the cholesterol pathway, we needed to investigate the precise morphology modification and the potential effect of MitoQ in preventing the autophagy mechanism. (Figure 5) [21,22].

After 12 h, the cellular control condition was represented by cells whose plasma membranes and inner membranes were intact. The organelles and nuclear envelope, indeed, exhibited a regular shape, and the chromatin showed normal architecture. The structure of the mitochondria was oval and/or elongated, mitochondrial cristae were well shaped, and the cisternae of the rough endoplasmic reticulum (RER) and other organelles were regular (Figure 5A). The framework in the presence of MitoQ was quite similar to the control cell culture. Elongated mitochondria were more frequently represented than oval ones (Figure 5D). The lovastatin treatment modified the cellular morphology. Vesicles were observed with degenerate membranes in the inner space (). Many cells showed light vesicles surrounded by membranes that seemed to be empty mitochondria. Several RER membranes arranged themselves in the three spacial directions among the mitochondria (Figure 5B). Most mitochondria were slightly swollen with degenerated cristae in the central area corresponding to a clear space in the mitochondrial matrix and well conserved at the periphery (Figure 5B). Some mitochondria exhibited not well-defined cristae.

MitoQ is able to rescue this alteration, and the cells appear in much better conditions than Lova-treated cells. Most mitochondria are normal (Figure 5C). Others are similar to those described in LOVA-treated cells (see above), but they show a much lower damage. In this case, we can observe small clear spaces in the mitochondrial matrix, corresponding to a low level of cristae degeneration (Figure 5C).

The microscopy analysis at 48 h showed cells quite similar to the 12 h control cultures, as the mitochondria exhibited regular morphology shapes, membranes and cristae (Figure 5E). The same occurred with the treatment of MitoQ, where the cells and their structures were analogous to the controls (Figure 5H). Only rare autophagy signs could be detected. A dramatic morphologic change was associated with lovastatin treatment after 48 h (Figure 5F)—many apoptotic and necrotic cells were present. Most cells were deeply modified. The cytoplasm was enriched with enormous vesicles surrounded by membranes containing scarce, unidentifiable material and organelles ascribable to mitochondria (Figure 5F). Other small vesicles with degraded material were present. Some mitochondria exhibited damaged cristae and inner empty areas; others appeared normal in structure (Figure 5F). Rare RER cisternae were visible. The potential protective role of MitoQ was not able to rescue the programmed cell death induced by statins. Rare apoptotic and necrotic cells were detectable. Most cells showed remarkable signs of autophagy [23] at different stages, as follows: (1) endoplasmic reticulum membranes surrounded mitochondria during the process of enclosing them into vesicles; and (2) large vesicles containing particulate material and/or damaged mitochondria showed evidence of the active autophagic process. Many normal mitochondria were present in the cytoplasm, and also other organelles were in very good conditions (Figure 5G).

### 2.5. The Blocking of the Mevalonate Pathway Induces Mitochondrial Dysfunction

To investigate the mitochondrial damage triggered by the blockage of the metabolic pathway, we analysed the mitochondrial membrane potential (ᐃΨM), commonly acknowledged as a marker of cellular health, by measuring Rhodamine 123 fluorescence (Rho) [24].

**Figure 6:**
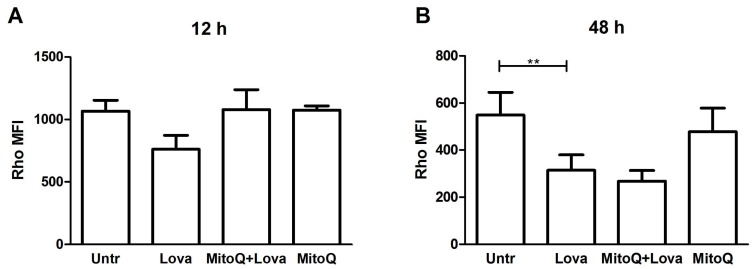
Mean fluorescent intensity (MFI) of Rhodamine 123 of three independent experiments at 12 h (**A**) and 48 h (**B**). Data are expressed as means and SDs (*n* = 3, ** *p* < 0.001 based on one-way ANOVA with Bonferroni post-test).

Lovastatin treatment increased mitochondrial dysfunction, as shown by a reduction inthe median fluorescence intensity (MFI) of Rho [25]. MitoQ pre-treatment restored, even if not in a significant manner, the MFI values on DAOY cells, only at the 12 h analysis (Figure 6A,B) [26].

The intermediate analyses of MFI at 24 and 36 h confirmed the progressive increase in mitochondrial dysfunction and the inability of MitoQ to protect mitochondria (Supplementary Data, Figure S2).

## 3. Materials and Methods

### 3.1. Reagents

If not differently specified, reagents were purchased from Sigma-Aldrich (St. Louis, MO, USA).

Lovastatin (Lova, Mevinolin from Aspergillus terreus) was dissolved in ethanol which did not exceed 0.01% of the final concentration in each well.

MitoQ was kindly provided by MP Murphy (MRC Mitochondrial Biology Unit, Cambridge, UK) and dissolved in ethanol. *N*-Acetyl-l-cysteine (Nac) was distilled in distilled water.

### 3.2. Cell Culture

DAOY (human-derived desmoplastic cerebellar medulloblastoma cell line), obtained from ATCC (ATCC^®^ HTB-186™, Manassas, VA, USA), was cultured in EMEM (Eagle’s Minimum Essential Medium) (Euroclone, Milan, Italy), supplemented with 10% foetal bovine serum (FBS, Euroclone), 2 mM glutamine and 1× solution of penicillin-streptomycin (Sigma-Aldrich). Twenty-four hours after seeding, cells were stimulated with pre-treatment of MitoQ (200 nM) for 1 h and then with Lova (10 μM). At the end of the incubation period, the supernatant was collected for the cytokine assay, while cells were used for impedance measurement, microscopy analysis and pelleted for the programmed cell death (PCD) or mean fluorescence intensity assay.

### 3.3. The xCELLigence System and Impedance Measurement

The effects of the Lova and MitoQ treatment were evaluated with the xCELLigence RTCA DP Instrument (Roche, Monza, Italy), which records the increase in electrical impedance due to the presence of adherent cells on the well bottom covered by microelectrodes. In real-time and without the addition of a label, the number, the morphology and the viability of attached cells were displayed as alterations in the impedance and converted into an adimensional parameter called the “cell index” (CI). A decrease in CI after adding a pharmacological compound could be due to a detachment or to the death of cultured cells. Briefly, for this analysis, 5 × 10^3^ DAOY were seeded in a 16 well E-plate in 200 µL of complete medium, and cultured in 5% CO_2_ at 37 °C. The cells were stimulated with MitoQ or NAC (10 mM) and after 1 h with Lova. The impedance was measured every 15 min; this experiment was reproduced in triplicate. The change in impedance was calculated by dividing the desired value by the value of the untreated condition.

### 3.4. Oil Red O Staining

A stock solution of 0.35% Oil Red O (BDH Chemicals, Poole, UK) in isopropanol was prepared, filtered twice using a 0.22 µm filter and diluted in double-distilled H_2_O (ddH_2_O; 3:2) prior to use. To detect cellular lipid accumulation, DAOY-treated cells were gently washed with PBS and fixed using 4% paraformaldehyde for 1 h at room temperature (RT). Subsequently, the cells were washed twice using ddH_2_O and stained with Oil Red O solution for 20 min at RT. To remove the background staining, the cells were washed for 5 min with 60% isopropanol solution. Lipid droplet accumulation was detected under a microscope.

### 3.5. Programmed Cell Death Assay

The programmed cell death (PCD) of DAOY was analysed by flow cytometry using Annexin V (A) and propidium iodide (PI) staining. Cells were stained with FITC-conjugated Annexin V and propidium iodide (Annexin V-FITC Apoptosis Detection Kit, Immunostep, Salamanca, Spain) following the manufacturer’s indications. Briefly, 12 and 48 h after stimulation, cells were harvested from the Petri dish with 0.5% trypsin-EDTA solution (Sigma Aldrich) and washed with PBS. Cells were stained with A and PI for 15 min, then washed with customer binding buffer.

Fluorescence was acquired with a FACSCalibur cell analyser and CellQuest software (version 5.1.1, Becton Dickinson, Milan, Italy) and then analysed with FlowJo software (version 7.6, Treestar, Inc., Ashland, OR, USA). Debris was excluded from the plot based on the scatter, while the apoptotic (A+/PI−; A+/PI+) and necrotic (A−/PI+) cells were characterised by the fluorescence emitted.

### 3.6. Immunohistochemistry

Cell pellets were fixed with 2.5% glutaraldehyde (Electron Microscopy Sciences, Hatfield, PA, USA) in 0.1 cacodylate buffer, at pH 7.3, for 1 h at room temperature. They were rinsed twice for 10 min in 0.1 cacodylate buffer and postfixed with 1% osmium tetroxide in the same buffer for 1 h at 4 °C [14]. Pellets were then dehydrated in ascending alcohols, treated with propylene oxide and embedded in araldite (Electron Microscopy Sciences). Ultrathin sections of the samples were cut on a Top Ultra 150 ultramicrotome (Pabish, Germany) and collected on 300-mesh copper grids (3.05 mm diameter). The grids were stained with uranyl acetate and lead citrate and examined at 80 kV using a Jeol JEM 100S transmission electron microscope.

### 3.7. Mean Fluorescence Intensity (MFI)

MFI was analysed by flow cytometry (Rhodamine 123, Sigma-Aldrich). Results are expressed as the median fluorescent intensity (MFI) of Rhodamine 123 from 3 independent experiments. Fluorescence was acquired with a FACSCalibur cell analyser and CellQuest software (version 5.1.1, Becton Dickinson) and then analysed with FlowJo software (version 7.6, Treestar).

### 3.8. Data Analysis

All results are expressed as means and standard deviations (SDs). Statistical significance was calculated using one-way or two-way analyses of variance (ANOVA), and Bonferroni post-test corrections were applied in the case of multiple comparisons. Analyses were performed using GraphPad Prism software (version 5.0) (Graph Pad Software, San Diego, CA, USA).

## 4. Conclusions

Our study focused on the fundamental roles of mitochondria-targeted drugs, such as MitoQ, in cellular protection after blocking of the cholesterol pathway [24]. To date, the etiopathogenesis of rare inborn diseases associated with the cholesterol pathway, as well as Mevalonic Aciduria or Smith–Lemli–Opitz Syndrome, still needs to be deeply studied [27]. The causative genes and symptoms associated with this disease are known but even though it is widely hypothesised, the role of the mitochondria in this pathogenesis remains unclear [20,27]. Data from the literature strongly supports mitochondrial involvement in inflammasome activation and all evidence from our study converges to establish that the mitochondria play a crucial role in the apoptotic mechanism, secondary to the blocking of the cholesterol pathway, and that the antioxidant MitoQ, administered as a preventive agent, could counteract cell damage induced by statins in the early stages (12 h post lovastatin stimulation). This protective role fades over time.

Recently, several studies have highlighted the potential protective effects of MitoQ in cardiac [28], respiratory [29] and renal pathologies [30].

Different experimental designs have shared evidence that MitoQ improves mitochondrial respiration so much that it is considered a promising drug that induces both autophagy and mitochondria-selective mitophagy. However, the mechanism by which MitoQ operates is unclear and is subject to controversial interpretation. Indeed, Sun C. et al. [31] claimed that MitoQ regulates autophagy by inducing a pseudo-mitochondrial membrane potential. However, Lyamzaev K.G. et al. contradicted this evidence. According to these authors, the decrease of mitochondrial membrane potential activates the mitophagy and is related to oxidative phosphorylation, which, in itself, represents an inducer of the mitochondrial autophagy process [32].

The results obtained show that the action of statins has effects on various cellular districts, including the mitochondria involved in signal transduction. In particular, biochemical blocking induces dramatic changes at the morphological and functional levels, such as the determination of programmed cell death. Microscopy evidence showed that the main effect of the MitoQ is to guarantee the morphologic conservation of the mitochondria and consequently, the protection of the cell itself.

The correct understanding of the mechanism by which MitoQ acts is undoubtedly a fundamental step towards enhancing protective effects on the mitochondria. Indeed, the temporary effects of MitoQ are not able to restore the cellular changes caused by the cholesterol pathway block, but further studies should be oriented towards identifying molecules able to strengthen the protective effects of MitoQ.

## Supplementary Materials

Supplementary materials can be found at http://www.mdpi.com/1422-0067/19/5/1523/s1.

## Abbreviations

PCD	programmed cell death
ᐃΨM	mitochondrial membrane potential
Rho	Rhodamine 123 fluorescence
