# Supplementary figures and images for: Neuronal Dysfunction Associated with Cholesterol Deregulation
Source: Int J Mol Sci. 2018 May 19;19(5):1523. doi: 10.3390/ijms19051523 (PMC5983599; doi:10.3390/ijms19051523)

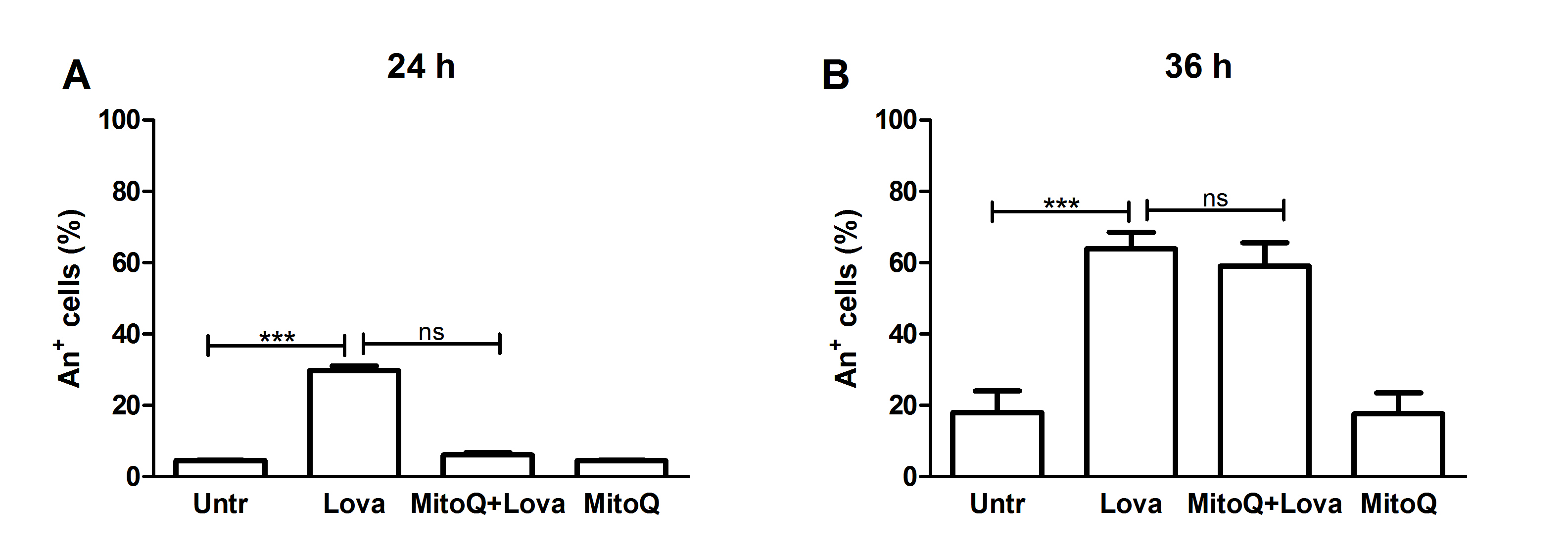

Supplement: Supplementary file 1 [file ijms-19-01523-s001.zip › AP 24 e 36 h.jpg]

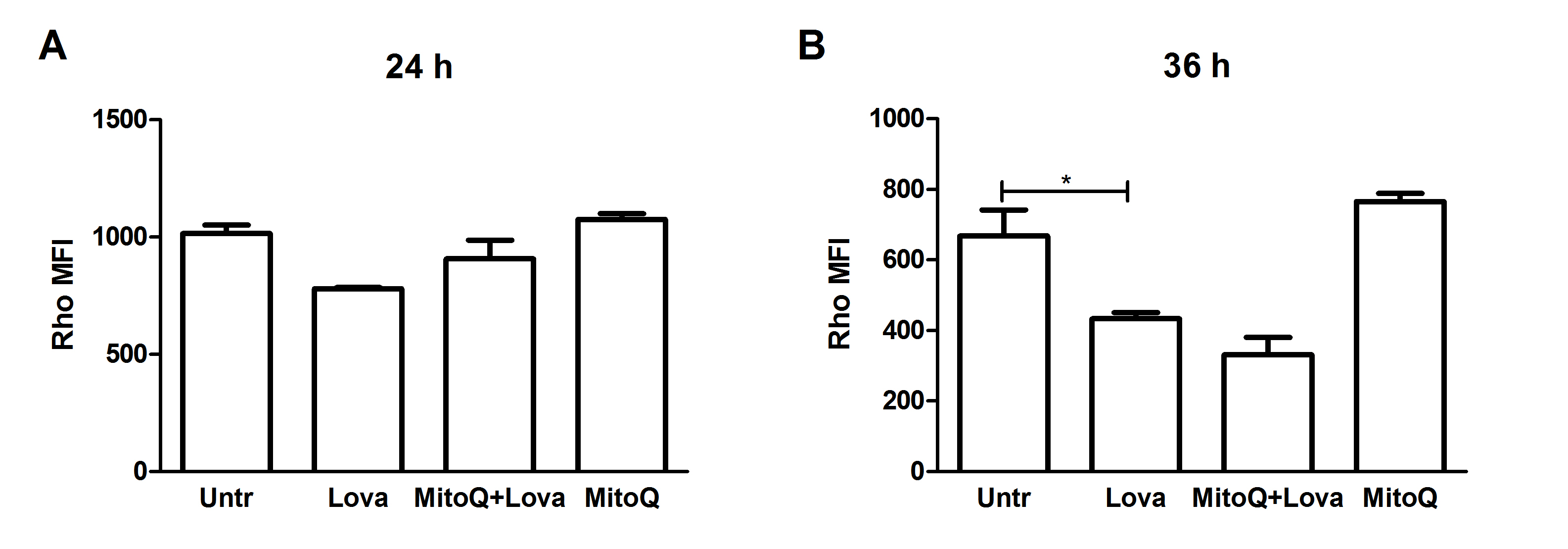

Supplement: Supplementary file 1 [file ijms-19-01523-s001.zip › Rho 24 e 36 h.jpg]
